# Amatoxin Intoxication and Wild Mushroom Poisoning: Current Advances in Diagnosis, Risk Stratification, and Clinical Management

**DOI:** 10.3390/toxins18050216

**Published:** 2026-05-03

**Authors:** Tsonka Dimitrova, Djeni Cherneva, Kaloyan Mihalev, Ivelin Iliev, Galina Yaneva, Svetlana Georgieva

**Affiliations:** 1 Department of Biology, Faculty of Pharmacy, Medical University of Varna, 9000 Varna, Bulgaria; tsonka.dimitrova@mu-varna.bg (T.D.); djeni.cherneva@mu-varna.bg (D.C.); 2 Department of Pharmaceutical Chemistry, Faculty of Pharmacy, Medical University of Varna, 9000 Varna, Bulgaria; ivelin.iliev@mu-varna.bg (I.I.); svetlana.georgieva@mu-varna.bg (S.G.)

**Keywords:** wild mushroom poisoning, amatoxin intoxication, mushroom toxic syndromes, diagnosis, risk stratification, clinical management, hepatotoxicity, molecular diagnostics

## Abstract

Wild mushroom poisoning remains a major medical and toxicological challenge worldwide because of the diversity of toxic compounds, the broad spectrum of clinical manifestations, and the risk of severe hepatic or renal injury. Early differentiation between self-limiting gastrointestinal syndromes and potentially fatal intoxications with progressive organ failure remain a central clinical challenge. This review examines recent advances in the diagnosis, risk stratification, and therapeutic management of wild mushroom poisoning, with amatoxin intoxication serving as the principal clinical focus. Selected evidence from other mushroom toxic syndromes is also included to support differential diagnosis, highlight syndrome-specific variability, and provide comparative clinical and methodological context. The recent literature indicates a shift from predominantly symptom-based diagnosis toward integrated models combining clinical evaluation, laboratory biomarkers, toxicological testing, and analytical and molecular methods. Liquid chromatography, mass spectrometry, immunoassays, and the molecular identification of fungal species have improved diagnostic precision, particularly in cases with uncertain exposure history or delayed presentation. Current management relies on early multimodal strategies including intensive supportive care, targeted pharmacological interventions, extracorporeal detoxification, and, in selected severe cases, liver transplantation. Overall, clinical outcome depends not only on toxin profile, but also on timely diagnosis, accurate early risk stratification, and prompt coordinated treatment. Future research should prioritize standardized diagnostic pathways, validated prognostic models, and clinically applicable treatment algorithms that support earlier escalation of care in severe mushroom intoxication.

## 1. Introduction

Poisonings with wild mushrooms represent a significant medical and toxicological problem in many regions of the world [1]. In addition to accidental foraging, a substantial proportion of mushroom poisoning cases arise from complex food chain-related pathways including misidentification during collection, unintentional mixing of edible and toxic species, and the distribution of contaminated wild mushrooms through informal or commercial food channels [1,2,3,4]. Reports from regions with high incidence, particularly in China and Southeast Asia, indicate that toxic species may enter the human food chain through local markets, street vendors, catering establishments, and household-level food preparation, where morphological similarity between edible and poisonous mushrooms contributes to frequent misclassification. Large-scale epidemiological analyses have identified species such as *Amanita exitialis*, *Amanita fuliginea*, *Galerina sulciceps*, and *Russula subnigricans* among the most common causes of severe and fatal intoxications, reflecting both regional biodiversity and patterns of consumption [3,5,6]. These observations highlight that mushroom poisoning is not only an issue of individual exposure, but also a broader public health problem influenced by food distribution systems, cultural practices, and variability in species recognition.

They present with an extraordinary variety of clinical manifestations, determined by the specific toxin profile of the ingested species [2]. Currently, more than fourteen clinical syndromes associated with toxic mushrooms have been described, with the spectrum of symptoms varying from mild gastroenteritis to severe conditions including acute liver or kidney failure, neurological disorders, and multiorgan dysfunction [7]. Early recognition of intoxication and the timely initiation of adequate therapy are of key importance for the clinical outcome.

The latency period between ingestion and symptom onset is traditionally used as an initial clinical orientation tool, with short-latency presentations generally associated with functional syndromes and long-latency presentations more often linked to organ toxicity. However, this distinction is not absolute and should be interpreted with caution [8,9].

The classic course of poisoning with amatoxin-containing mushrooms is described by four successive phases [8]. Initially, a latent phase occurs, in which there are no clinical symptoms and patients often do not associate subsequent manifestations with the previous consumption of mushrooms. This is followed by the gastrointestinal phase, characterized by watery diarrhea, recurrent vomiting, abdominal pain, and the risk of severe dehydration and hypovolemic shock. The third stage is the so-called phase of apparent convalescence, in which symptoms temporarily subside while toxic effects on the liver and kidneys progress. In the final phase, multiorgan failure develops, accompanied by severe elevation of liver enzymes, coagulopathy, metabolic acidosis, hypoglycemia, and hepatic encephalopathy. This dynamic and often deceptive clinical progression, particularly the transient improvement phase preceding severe organ toxicity, presents a significant diagnostic challenge and may delay the recognition of life-threatening intoxication.

In this context, accurate and timely diagnostic strategies are essential for the early identification and risk stratification of mushroom poisoning. In recent years, significant progress has been made in the diagnosis of these intoxications [9]. In addition to traditional toxicological and biochemical studies, molecular genetic methods, including polymerase chain reaction and next-generation sequencing, are increasingly being used, which allow for the precise identification of mushroom species and their toxins [10]. New analytical techniques are also being developed for the direct detection of toxins in biological samples [11]. In parallel, digital technologies such as mobile applications for mushroom recognition and artificial intelligence-based models are increasingly being explored as adjunctive tools for early triage and risk assessment in suspected mushroom poisoning. However, their diagnostic reliability remains variable, and they should not be regarded as substitutes for expert identification, toxicological evaluation, or clinical judgment [12].

Significant progress has also been made in therapeutic approaches. In addition to standard detoxification and supportive care, current clinical practice includes the use of specific antidotes such as silibinin and N-acetylcysteine as well as various extracorporeal blood purification techniques including hemoperfusion, hemodialysis, and molecular adsorption systems [13]. In the event of fulminant liver failure, liver transplantation remains the only life-saving therapeutic option.

Given the wide spectrum of toxic syndromes associated with wild mushroom poisoning, this review adopts a focused analytical framework centered on amatoxin intoxication. This approach is justified by the disproportionately high morbidity and mortality associated with amatoxin-containing species, their global epidemiological relevance, and the complex clinical course characterized by delayed onset and rapid progression to acute liver failure. In addition, amatoxin poisoning represents the most extensively studied and clinically challenging form of mushroom toxicity, making it a suitable reference model for discussing diagnostic and therapeutic strategies. At the same time, selected evidence from non-amatoxin syndromes is incorporated where it provides clinically relevant comparative insights, particularly in the context of differential diagnosis, early risk stratification, or methodological advances applicable across different toxicological profiles. Such inclusion is not intended to provide a comprehensive overview of all mushroom poisoning syndromes, but rather to contextualize key diagnostic and management challenges within a broader toxicological framework.

Within this structure, the present review aims to critically evaluate current advances in the diagnosis, risk stratification, and clinical management of wild mushroom poisoning, with particular emphasis on the applicability and limitations of emerging diagnostic tools and therapeutic strategies in real-world clinical settings.

## 2. Materials and Methods

### 2.1. Search Strategy

This review was conducted as a structured narrative review. A comprehensive literature search was conducted across PubMed/MEDLINE, EMBASE, and Web of Science for articles published between January 2008 and March 2025. The lower time limit of January 2008 was selected to focus the review on contemporary evidence reflecting the major developments of the past two decades in analytical toxicology, molecular diagnostic methods, prognostic assessment, extracorporeal support techniques, and modern critical care management of severe mushroom poisoning. A formal flow diagram and quantitative study selection process were not applied, as the review was conducted using a structured narrative approach rather than a systematic review framework. The search strategy combined the following terms using Boolean operators: (“mushroom poisoning” OR “mushroom intoxication” OR “wild mushroom toxicity” OR “fungal poisoning” OR “*Amanita phalloides*” OR “amatoxin”) AND (“mushroom toxin diagnosis” OR “risk stratification” OR “severity assessment” OR “mass spectrometry mushroom toxin” OR “LAMP mushroom identification” OR “silibinin” OR “extracorporeal detoxification” OR “acute liver failure” OR “liver transplantation”). Where appropriate, the NOT operator was applied to exclude irrelevant records.

A total of 668 records were identified through database searching. After the removal of 493 duplicates, 175 records remained for title and abstract screening. Following screening, 60 articles were assessed for full-text eligibility.

### 2.2. Inclusion and Exclusion Criteria

Studies were included if they reported original data or analytical findings on the diagnosis, risk stratification, or clinical management of wild mushroom poisoning in humans, were published in peer-reviewed journals, and were available as full-text articles.

Studies were excluded if they focused exclusively on mushroom cultivation, nutrition, or non-toxic species without clinical relevance, were published as conference abstracts, letters, or editorials without original data, or involved animal models only without direct clinical translational relevance.

### 2.3. Data Synthesis

Given the heterogeneity of the available literature and the clinical orientation of the present work, this review was conducted as a structured narrative review rather than as a formal systematic review or meta-analysis. Accordingly, studies were selected based on their clinical relevance, methodological robustness, and direct applicability to the main aims of the review, namely diagnosis, risk stratification, and the clinical management of wild mushroom poisoning. Priority was given to studies providing clinically actionable evidence, including cohort studies, systematic reviews, and well-documented case series. Experimental, analytical, and methodological studies were also included when they offered important insight into emerging diagnostic or therapeutic approaches. Studies addressing non-amatoxin syndromes were incorporated selectively, particularly when they illustrated clinically relevant variability, syndrome-specific diagnostic or therapeutic challenges, or methodological approaches potentially applicable to severe mushroom intoxications more broadly.

The included studies were grouped thematically into three principal domains: (i) laboratory and toxicological diagnosis; (ii) molecular and emerging analytical methods; and (iii) therapeutic management including pharmacological, extracorporeal, and surgical interventions.

Studies included in the summary tables were additionally appraised for their level of evidence based on study design, using a modified Oxford Center for Evidence-Based Medicine (CEBM) framework adapted to the study designs encountered in the clinical toxicology literature: High—systematic reviews or meta-analyses of randomized controlled trials or large prospective cohort studies; Moderate–High—systematic reviews of observational studies or well-designed prospective cohort studies; Moderate—retrospective cohort studies and comparative analytical studies with adequate sample sizes; Low–Moderate—small retrospective or cross-sectional studies and reference database validation studies; Low—case reports, case series, methodological or analytical validation studies, and narrative reviews.

## 3. Clinical Characteristics of Wild Mushroom Poisoning: Latency-Based Classification and Amatoxin Toxidrome

The symptoms of poisoning can be divided into four main stages:

First (lag) stage. The latency period is relatively long, averaging eight to ten hours, although in rare cases it may extend to 24–36 h. There are no signs of intoxication, and, proportionately to the length of the period, patients often fail to associate subsequent symptoms with mushroom ingestion that occurred one to two days earlier.

Second (gastrointestinal) stage. It lasts up to two days and includes: watery diarrhea, abdominal pain, profuse and recurrent vomiting, resulting in dehydration, hypovolemic shock, and hypoglycemia. Failure to recognize this clinical stage may result in misdiagnosis of acute gastroenteritis and premature discharge.

Third (apparent convalescence) stage. The gastrointestinal symptoms slowly diminish, however, toxic injury to the liver and kidneys progresses, and the serum levels of aspartate aminotransferase, alanine aminotransferase, and lactate dehydrogenase rise rapidly. Coagulopathy may lead to internal bleeding.

Fourth (multiorgan failure) stage. Numerous deteriorating metabolic parameters show that progressive toxin-induced injury leads to marked elevations in liver transaminases, lactate dehydrogenase, and bilirubin, together with coagulation abnormalities, metabolic acidosis, hypoglycemia, and encephalopathy as manifestations of increasingly extensive liver and kidney damage [14].

According to the interval between mushroom ingestion and symptom onset, mushroom poisoning can be broadly classified into short-latency and long-latency presentations. In general, poisonings with a latency of less than six hours are more commonly associated with functional, neurotoxic, or gastrointestinal syndromes, whereas those with a latency exceeding six hours are more often linked to organ-damaging syndromes including phalloides, gyromitra, orellanus, and rhabdomyolysis syndromes [1]. However, this temporal distinction should be interpreted with caution, as short-latency presentations are not invariably benign, and early symptoms may occasionally coexist with or precede the later manifestation of severe delayed toxicity. For example, severe muscarinic toxidrome caused by *Clitocybe* or *Inocybe* species, although typically presenting within 30 min to 2 h of ingestion, may be life-threatening due to bronchospasm, excessive secretions, and cardiovascular compromise, particularly in vulnerable populations.

Up to 14 syndromes caused by wild mushroom poisoning, depending on the species, toxins, and amount ingested, are described [15]. The clinical presentation of these common poisonings ranges from benign symptoms of generalized gastrointestinal disorders to potentially devastating clinical manifestations such as liver and kidney failure as well as neurologic sequelae. These include acute gastroenteritis, hallucinations, cholinergic toxicity occurring within 30 min (abdominal cramping, diaphoresis, salivation, lacrimation, bronchospasm, bronchorrhea, and bradycardia), headache, nausea, vomiting, flushing, tachycardia, liver toxicity, nephrotoxicity, seizures as well as vertigo, somnolence, palpitations, dysrhythmias, rhabdomyolysis, methemoglobinemia, hemolysis, erythromelalgia, dermatitis, and rarely, hypotension. These clinical patterns highlight the complexity and variability of mushroom intoxications and underscore the importance of early syndrome recognition as a critical step guiding subsequent diagnostic and therapeutic decision-making.

## 4. Diagnosis of Wild Mushroom Poisoning, with Emphasis on Amatoxin-Related Intoxication

### 4.1. Laboratory Diagnosis of Wild Mushroom Intoxications

The laboratory diagnosis of wild mushroom intoxication relies on a combination of clinical history, biochemical markers, toxicological detection of mushroom toxins, and increasingly, molecular and computational methods. Laboratory diagnostic approaches can be broadly divided into three groups: (i) detection of mushroom toxins in biological samples; (ii) evaluation of biochemical markers reflecting organ injury and prognosis; and (iii) emerging computational and analytical tools supporting clinical decision-making.

Given the heterogeneity of the available evidence, the findings presented in this section are interpreted in the context of their respective study design and corresponding level of evidence. Particular attention is given to distinguishing between higher-level evidence derived from cohort studies and systematic reviews, and lower-level evidence from case reports and small case series.

Although the most clinically validated diagnostic pathways remain centered on amatoxin-related poisoning, selected studies on other mushroom syndromes are also relevant because they illustrate broader advances in species identification, toxin detection, and syndrome-oriented diagnostic differentiation.

The diagnosis of *A. phalloides* poisoning is based on the history of recent wild mushroom ingestion followed by gastrointestinal symptoms, typical time course and laboratory markers, and confirmed by mycological or toxicological analysis [16].

Quantitative determination of amanitins in the blood and urine of 698 patients with suspected *A. phalloides* poisoning in Slovakia using the original enzyme-linked immunosorbent assay kit was performed [17]. The urinary amanitin examination correlated with the severity of poisoning in the range of 6–47 h after mushroom ingestion without any false negativity, while the serum assay showed no diagnostic value. These observations highlight the importance of timing and analytical methodology in toxin detection, underscoring that diagnostic sensitivity is highly dependent on both sample type and the interval between exposure and testing.

In a retrospective machine learning study involving 567 critically ill adult patients with mushroom poisoning from five primary care hospitals and facilities in Enshi, Hubei Province, China, four algorithms were applied to develop an early triage model based on clinical indicators [18]. The training and the test cohorts included 322 and 245 patients, respectively. The extreme gradient boosting displayed the best discriminative ability in fivefold cross-validation (area under the curve of 0.83; between 0.77 and 0.90 at a confidence interval of 95%) and in the test set (area under the curve of 0.90; between 0.83 and 0.96 at confidence interval of 95%). In the test set, this 14-factor model had a sensitivity of 0.93 (between 0.81 and 0.99 at confidence interval of 95%) and a specificity of 0.79 (between 0.73 and 0.85 at confidence interval of 95%), while the physicians’ assessment had a sensitivity of 0.86 (between 0.72 and 0.95 at confidence interval of 95%) and a specificity of 0.66 (between 0.59 and 0.73 at confidence interval of 95%). This model may support early treatment selection and referral decisions and could potentially contribute to improved clinical outcomes. While these findings suggest strong predictive performance, the retrospective design and single-country cohort limit generalizability, and external validation in diverse clinical settings remains necessary.

In a retrospective cohort study conducted between January 2009 and December 2018, 105 patients with *A. phalloides* intoxication treated at two hospitals of the China Medical University in Liaoning were evaluated using laboratory markers and several clinical scoring systems including Child–Turcotte–Pugh, Sequential Organ Failure Assessment, Liver Injury and Failure Evaluation, Chronic Liver Failure–Organ Failure score, King’s College criteria, Model for End-Stage Liver Disease, and Platelet–Bilirubin–Albumin, all assessed within 24 h of admission [19]. Multivariate logistic regression analysis showed that an international normalized ratio above 3.6 (AUC 0.941) and plasma ammonia above 95.1 μmol/L (AUC 0.805) were independently associated with mortality, while a Chronic Liver Failure–Organ Failure score above 9 within 24 h demonstrated excellent predictive performance and outperformed the other evaluated scoring systems [19]. However, the retrospective design of the study limits the strength of causal inference, and the findings should be interpreted as prognostic associations rather than definitive evidence for clinical decision-making.

A descriptive retrospective cross-sectional study involving 65 patients hospitalized for wild mushroom intoxication at Razi Hospital in Qaemshahr, Mazandaran, Iran, identified statistically significant abnormalities in alanine aminotransferase, international normalized ratio, prothrombin time, and partial thromboplastin time (*p* = 0.003, *p* = 0.006, *p* = 0.035, and *p* = 0.050, respectively) [20]. These findings provide supportive but limited evidence because of the relatively small sample size and the observational nature of the study.

The review of more than 167 articles devoted to wild edible mushrooms and published during the past 20 years outlines the analytical tools and data analysis methods for the identification and quality evaluation of these species, their origin, and mineral elements [21]. Five macroscopic, microscopic, and molecular identification techniques are used. Chromatography and spectroscopy technology combined with chemometrics are applied for the qualitative and quantitative investigation of mushrooms and the evaluation of mushroom quality. Deep learning shows its advantages in image recognition and prediction.

These findings indicate a shift from reliance on isolated laboratory parameters toward integrated diagnostic models that combine clinical, biochemical, and analytical data. This evolving approach provides the basis for the subsequent development of advanced molecular and instrumental diagnostic techniques.

### 4.2. Instrumental Diagnostic Methods of Wild Mushroom Intoxications

#### 4.2.1. Molecular Diagnostic Methods

Molecular diagnostic studies in wild mushroom poisoning extend beyond amatoxin-producing species and are included here to demonstrate the broader applicability of rapid species-identification technologies in toxicology. Although these assays were developed for different toxic mushrooms and clinical syndromes, together, they illustrate transferable diagnostic principles, particularly with respect to sensitivity in processed samples, resistance to sample degradation, and the ability to detect target species in mixed biological or food matrices.

Two rapid and sensitive detection methods for *A. citrinoannulata* have been reported, based on colorimetric and real-time loop-mediated isothermal amplification using primers specifically targeting the internal transcribed spacer region [22]. Both assays demonstrated high analytical sensitivity, with a detection limit of 0.2 ng of *A. citrinoannulata* DNA, and showed no cross-reactivity with 41 non-target mushroom species. The entire workflow was completed within 40 min using standard laboratory equipment, with results interpretable by naked-eye visualization. Importantly, both methods successfully identified the target species in fresh, cooked, and emetic samples containing as little as 1% *A. citrinoannulata*, underscoring their potential utility in clinical and forensic settings where sample quality is often compromised. The authors suggest that wider implementation of these methods may facilitate earlier species identification and improve clinical decision-making in selected settings.

Loop-mediated isothermal amplification and hyperbranched rolling circle amplification methods are applied to detect and distinguish different lethal *Amanita* species [23]. The loop-mediated isothermal amplification-based assay discriminates the introclade rather than the intraclade lethal *Amanita* species, while the hyperbranched rolling circle amplification-based one discriminates both species. The universal loop-mediated isothermal amplification primers were positive for ten lethal *Amanita* species, section *Phalloideae*, and negative for 16 *Amanita* ones outside this section. The detection limits of these two rapid, specific, sensitive and low-cost methods were 10 pg and 1 pg of genomic DNA per reaction, respectively.

A visual, rapid, accurate, sensitive, and low-cost method of *Gyromitra infula* identification based on loop-mediated isothermal amplification and with a designed set of specific primers was developed [24]. The sensitivity assay indicated a minimum concentration of genomic DNA detected by this method of 1 ng/μL. The content of *G. infula* as low as 1% was successfully detected when mushroom samples were boiled and digested in artificial gastric juice. The examination was completed within 90 minutes and results were directly interpretable by naked-eye visualization.

A set of loop-mediated isothermal amplification assays based on a real-time fluorescence and a visualization method for *Russula senecis* detection was developed and the visual reaction system was optimized to shorten the reaction time [25]. Both methods detected as low as 3.2 pg of genomic DNA. Fried and digested mushrooms were used to validate the proposed loop-mediated isothermal amplification method, and mushroom mixtures with as low as 1% of the target species were successfully identified, indicating that these assays have good applicability for clinical sample detection and forensic identification.

A matrix-assisted laser desorption/ionization time of flight mass spectrometry reference database was created and internally validated for 15 common *Amanita* mushroom species in France [26]. This database was challenged with 38 *Amanita* mushroom specimens from four French locations by means of a free online application for its spectra identifications. Decayed portions of *A. phalloides* mushrooms were properly identified using this technique. However, its reliance on specialized instrumentation limits its availability in emergency clinical settings.

A loop-mediated isothermal amplification method for the quick and accurate detection of *Omphalotus japonicus*, a major toxic mushroom in Japan, was developed [27]. The amplification occurs within 60 min, and wild mushroom presence or absence is confirmed within two hours, including the DNA extraction protocol. This method does not display any cross-reactivity with 13 edible mushrooms species. It has a high specificity toward *O. japonicus* and sufficient detection sensitivity even in a mixed mushroom sample containing 1% of this mushroom only.

Four primer sets for targeting *Psilocybe cubensis* DNA through multi-locus identification by means of real-time polymerase chain reaction with high-resolution melting were designed [28]. The target markers include the largest subunit of RNA polymerase II, psilocybin-related phosphotransferase gene, glyceraldehyde 3-phosphate dehydrogenase, and translation EF1α. The melting temperatures of these four markers of *P. cubensis* are 87.93 ± 0.12 °C, 82.21 ± 0.14 °C, 79.72 ± 0.12 °C and 80.11 ± 0.19 °C, respectively. A significant high-resolution melting characteristic was demonstrated with a low concentration of 62.5 pg/µL DNA sample. This analytical method quickly and specifically distinguishes *P. cubensis* from other mushroom species.

*Cantharocybe virosa* (Agaricales, Hygrophoraceae), a suspected gastrointestinal toxin-containing wild mushroom, was identified not only by means of DNA sequence analyses of the internal transcribed spacer region and the large subunit of nuclear ribosomal DNA, but also through liquid chromatography-quadrupole time-of-flight-mass spectrometry in 39 patients in Thailand [29].

These studies indicate that the value of rapid molecular identification extends beyond amatoxin-producing mushrooms and may be especially relevant in syndromes where early species-level distinction can refine differential diagnosis before organ-specific toxicity becomes fully apparent. These molecular identification approaches collectively demonstrate a fundamental shift toward species-level precision in mushroom toxicology, with consistent performance characteristics across different toxic taxa. The convergence on isothermal amplification methods (LAMP/HRCA) reflects their practical advantages in emergency settings: rapid turnaround times (40–90 min), minimal equipment requirements, and resistance to sample degradation from cooking or gastric processing. Notably, all validated assays achieved detection limits in the picogram to nanogram range and maintained specificity when tested against large panels of non-target species, suggesting that molecular identification has reached analytical maturity for clinical implementation. The ability to detect target DNA in processed and mixed samples addresses a critical gap in traditional morphological identification, where sample integrity is often compromised.

These characteristics make LAMP-based approaches among the most promising candidates for rapid deployment in time-sensitive clinical scenarios. However, the clinical impact of these advances remains contingent on integration with toxicological workflows and availability in time-sensitive clinical environments.

It should be noted that most of these findings are derived from methodological and validation studies rather than clinical outcome-based investigations, and therefore primarily support diagnostic feasibility rather than direct clinical effectiveness.

Despite their high analytical sensitivity and specificity, the clinical applicability of these molecular identification methods in acute toxicological settings remains variable. Techniques such as LAMP and related isothermal amplification approaches offer practical advantages, including rapid turnaround time and minimal equipment requirements, and may be suitable for near-point-of-care implementation in selected settings. In contrast, methods requiring advanced instrumentation, such as MALDI-TOF MS and high-resolution PCR-based platforms, are currently more applicable to specialized laboratory or reference center environments rather than real-time emergency decision-making. Furthermore, a significant proportion of the available studies represent analytical validation or proof-of-concept investigations rather than clinical outcome-based research. As a result, while these technologies substantially improve species-level identification, their direct impact on acute clinical management and patient outcomes remains to be clearly established.

While molecular diagnostic approaches significantly enhance species-level identification, their clinical utility remains dependent on timely availability and integration into acute care workflows, limiting their role as primary decision-making tools in emergency settings.

#### 4.2.2. Toxicological Detection Methods

A highly sensitive and automated quantification magnetic bead-based chemiluminescence immunoassay for the early and rapid diagnosis of wild mushroom poisoning was established [30]. The limits of detection for phallotoxins were 0.010 ng/mL in human serum and 0.009 ng/mL in human urine. Recoveries ranged from 81.6% to 95.6% with a variation coefficient <12.9%. The advantages of this method, such as high sensitivity, repeatability, and stability, are due to the use of magnetic beads as immune carriers, chemiluminescence as a detection signal, and an integrated device to automate the whole diagnostic process.

A strategy to reduce analysis time by focusing on two sets of analytes (i.e., for biomarkers of late-onset syndromes such as phalloides syndrome or the syndrome after castor bean intake and for biomarkers of early-onset syndromes such as pantherine-muscaria syndrome and muscarine syndrome indicating toxic mushroom or *Ricinus communis* ingestions) has been developed [31]. These two analyses in urine samples are based on hydrophilic-interaction liquid chromatography coupled with high-resolution mass spectrometry. The first method was validated for ricinine, α-amanitin, and β-amanitin and the second one for muscarine, muscimol, and ibotenic acid according to the specifications for qualitative analytical methods. The applicability was tested using ten urine samples from patients after suspected wild mushroom poisoning. The analytes α-amanitin, β-amanitin, muscarine, muscimol, and ibotenic acid were successfully identified. Psilocin-O-glucuronide was identified in two samples, and unambiguously distinguished from bufotenine-O-glucuronide. This new method is more labor-, time-, and cost-efficient as well as more robust and more sensitive.

This broader analyte panel is particularly informative because it reflects a clinically important reality: patients often present before the toxic syndrome has been clearly classified, and diagnostic workflows must therefore accommodate both late-onset hepatotoxic syndromes and early-onset neurocholinergic or neurotoxic presentations.

A liquid chromatography-tandem mass spectrometry method for the detection of α-amanitin, β-amanitin, and γ-amanitin in urine using isotopically labeled 15N10-α-amanitin and a modified amanitin methionine sulfoxide synthetic peptide as the internal standard was developed [32]. α-Amanitin precision and accuracy in pooled urine was ≤5.49% and between 100 and 106%, respectively, with a reportable range between 1 ng/mL and 200 ng/mL. β-Amanitin and γ-amanitin were most accurately quantitatively estimated in pooled urine using external calibration, revealing a precision ≤17.2% and an accuracy between 99 and 105% with calibration ranges between 2.5 ng/mL and 200 ng/mL and between 1.0 ng/mL and 200 ng/mL, respectively. Although amanitin intoxication can be identified from a wild mushroom ingestion history and timing of symptom onset, this new method represents a valuable clinical tool to confidently diagnose the exposure to α-amanitin, β-amanitin, and γ-amanitin.

A quantitative analysis of the concentration of ustalic acid, one of the primary toxic components in *Tricholoma ustale*, by liquid chromatography-tandem mass spectrometry preceded by purification using solid-phase extraction, was carried out [33]. This method is extremely sensitive. The limits of quantitation calculated at a signal-to-noise ratio of 10 were 10 ng/g (Shiitake mushroom) and 0.40 ng/g (miso soup). The accuracies of quantitation in these two samples ranged between 99.8% and 105% and between 98.8% and 102%, respectively. In leftover mushroom samples from a food poisoning case, ustalic acid was detected at 0.57–3.7 μg/g.

The analytical evolution in mushroom toxin detection reflects a strategic focus on syndrome differentiation rather than comprehensive toxin screening. The development of dual-panel approaches targeting both early-onset neurotoxic markers (muscarine, muscimol, ibotenic acid) and late-onset hepatotoxic compounds (amanitins, ricinine) represents a clinically oriented analytical strategy that mirrors the temporal decision-making requirements in emergency medicine. The consistent achievement of sub-nanogram detection limits across different platforms (LC-MS/MS, immunoassays, chromatographic methods) indicates that analytical sensitivity is no longer a limiting factor in toxin confirmation. Instead, the primary challenges appear to be standardization across laboratories and integration into clinical workflows where rapid results directly influence patient management decisions within critical time windows.

These developments highlight the increasing analytical capability of toxin detection methods; however, their clinical impact ultimately depends on rapid accessibility and their alignment with time-sensitive therapeutic decision-making.

#### 4.2.3. Emerging Technologies

In a serum-based untargeted metabolomics study, ultrahigh-performance liquid chromatography–quadrupole time-of-flight tandem mass spectrometry (UHPLC-QTOF-MS/MS) was used to compare 61 patients with amatoxin poisoning and 61 matched healthy controls, revealing 33 differential metabolites, of which 15 were upregulated and 18 were downregulated in the patient cohort [34]. These metabolites were primarily associated with lipid and amino acid metabolism pathways, including glycerophospholipid and sphingolipid metabolism as well as the tyrosine, arginine, proline, and phenylalanine-tryptophan biosynthesis pathways, all of which appear to play a role in the pathophysiology of amatoxin intoxication. Eight metabolic markers achieved satisfactory diagnostic accuracy in discriminating patients from healthy controls: glycochenodeoxycholate-3-sulfate, 11-oxo-androsterone glucuronide, neomenthol-glucuronide, dehydroisoandrosterone 3-glucuronide, glucose 6-phosphate, lanthionine ketimine, glycerophosphocholine, and nicotinamide ribotide. Of these, 11-oxo-androsterone glucuronide, glucose 6-phosphate, and glycochenodeoxycholate-3-sulfate showed a positive correlation with the degree of amatoxin-induced hepatic injury, suggesting their potential value as prognostic biomarkers in addition to their diagnostic utility [34].

The accuracy of three iPhone™ and Android™ mushroom identification applications such as Picture Mushroom (Next Vision Limited©), Mushroom Identificator (Pierre Semedard©), and iNaturalist (iNaturalist, California Academy of Sciences©) was compared using digital photographs of 78 specimens in Melbourne, Australia between 2020 and 2021 [35]. Picture Mushroom was the most accurate application and correctly identified 49% of the specimens followed by Mushroom Identificator and iNaturalist, each with an accuracy of 35%. Picture Mushroom correctly identified 44% of poisonous mushrooms followed by iNaturalist and Mushroom Identificator with accuracies of 40% and 30%, respectively. However, Mushroom Identificator correctly identified more specimens of *Amanita phalloides* (67%) than Picture Mushroom (60%) and iNaturalist (27%).

It should be noted that despite their potential as supportive tools, the relatively limited accuracy of these applications highlights the need for cautious interpretation and expert verification. This limitation has important public health implications. Smartphone-based identification tools may create false reassurance and delay toxicological consultation when probabilistic matches are misinterpreted as confirmation of safety. Accordingly, such tools should be framed, at most, as supplementary educational aids rather than decision-making instruments for mushroom consumption. This caution is consistent with public health recommendations issued by the French Agency for Food, Environmental and Occupational Health and Safety (ANSES), which explicitly advises against consuming mushrooms identified solely by smartphone recognition applications because of the high risk of error and recommends expert verification in cases of uncertainty. Key characteristics of the diagnostic studies reviewed in this section, including study design, level of evidence, and main findings, are summarized in Table 1.

## 5. Therapeutic Management of Wild Mushroom Poisoning, with Emphasis on Amatoxin-Related Severe Toxicity

### 5.1. General Principles of Management

Current management of *A. phalloides* intoxication consists of detoxification procedures, supportive measures, drug administration, and therapy in the specialized intensive care unit in the case of acute liver failure [16]. Urgent liver transplantation is the only life-saving option in selected patients with acute liver failure. The available evidence supporting these approaches is largely derived from observational studies, retrospective analyses, and expert consensus rather than randomized controlled trials.

Current European and international toxicology recommendations emphasize early supportive care, prompt administration of activated charcoal, and the use of silibinin and/or N-acetylcysteine as first-line pharmacological interventions in suspected amatoxin poisoning. Early referral to specialized centers and consideration of liver transplantation in cases of acute liver failure are also consistently recommended components of management [1,2].

To our knowledge, no clinical practice guideline specifically dedicated to the management of mushroom poisoning has been issued by the European Association of Poisons Centers and Clinical Toxicologists (EAPCCT) or the European Society of Intensive Care Medicine (ESICM). However, two published guideline documents provide relevant recommendations applicable to severe amatoxin-induced liver injury. The European Association for the Study of the Liver (EASL) Clinical Practical Guidelines on the management of acute (fulminant) liver failure recommend early transfer to specialized transplant-capable centers and the use of established prognostic models, including King’s College criteria, for transplantation decision-making in this context [36]. More recently, the American College of Gastroenterology (ACG) Acute Liver Failure Guidelines, developed using the Grading of Recommendations, Assessment, Development and Evaluation (GRADE) methodology, specifically recommend the prompt initiation of intravenous silibinin dihemisuccinate in patients with acute liver failure (ALF) due to mushroom poisoning, with intravenous penicillin G proposed as an alternative when silibinin is unavailable; this recommendation carries a conditional grading with very low quality of evidence, reflecting the absence of randomized controlled trials in this indication [37]. Both documents acknowledge the rarity of the condition and the predominance of observational data as fundamental constraints on the strength of achievable recommendations.

The management of amatoxin poisoning should be guided by the degree of diagnostic certainty and the patient’s clinical presentation. Treatment should be initiated promptly, including in asymptomatic individuals with a credible history of amatoxin exposure, because the absence of early symptoms does not exclude significant toxin absorption [38]. When the ingested species cannot be identified with certainty, supportive measures should be commenced without delay while species identification is pursued in parallel. The primary therapeutic objectives are hemodynamic stabilization and correction of fluid and electrolyte deficits. Elimination of absorbed amatoxins is pursued through a combination of activated charcoal administration and extracorporeal methods including hemodialysis, hemoperfusion, and plasmapheresis. More recently, the molecular adsorbent recirculating system (MARS) has been employed as an extracorporeal purification strategy, facilitating the transfer of toxic metabolites from the systemic circulation into a dialysate compartment across a selective membrane. The efficacy of all extracorporeal detoxification modalities is time-dependent and substantially diminished when initiation is delayed. Pharmacological intervention, encompassing both naturally derived and synthetic agents, constitutes an additional and integral component of the therapeutic strategy.

Treatment for amatoxin intoxication has four main options: (i) volume replacement therapy including electrolytes; (ii) toxin binding and elimination by hemodialysis or activated charcoal; (iii) antidote therapy with penicillin G, silibinin, and N-acetylcysteine; and (iv) treatment for liver failure including liver transplantation [1].

At present, there is no specific detoxification drug for α-amanitin, the main lethal toxin in *Amanita* mushroom, and thus the clinical treatment mainly focuses on symptomatic and supportive therapy [39].

Taken together, these principles emphasize that early recognition, prompt initiation of therapy, and timely referral to specialized centers are the cornerstone determinants of clinical outcome in severe mushroom poisoning.

### 5.2. Antidote and Pharmacological Therapy

Currently, there is no international consensus on the management of *A. phalloides* poisoning. Antidotes with antioxidant properties remain the most widely used therapeutic agents, suggesting the predominant role of oxidative stress in the pathophysiology of this intoxication [40]. The partially elucidated mechanisms of action may reveal a suitable target for the development of an antidote. The contemporary knowledge on amanitins and the latest advances that allow for the proposal of new innovative and effective therapeutics are reviewed.

Recently, a wide range of medications such as penicillin G, silibinin, N-acetylcysteine, thioctic acid, corticosteroids, ceftazidime, cimetidine, vitamin C, vitamin E, insulin, glucagon, and human growth hormone have been used either alone or in combination for the management of *A. phalloides* intoxication [14].

Between 2004 and 2020, 129 patients with confirmed *A. phalloides* intoxication received the full treatment protocol with antidotes of penicillin G plus silibinin, while 12 patients were treated with silibinin only in Slovakia [17]. There were two lethal cases in the first group due to acute kidney injury in the early stages of poisoning as well as four deaths in the second group caused by fulminant liver failure and intracranial hemorrhage. One patient in the second group underwent liver transplantation. Treatment failure was statistically significantly more common in the second than in the first group (41.67% vs. 1.57%; *p* = 0.00058).

The results from a systematic review of 13 studies with a total of 506 patients with amatoxin poisoning and N-acetylcysteine treatment, which were retrieved from the PubMed, EMBASE, CENTRAL, and SinoMed databases up to 31 August 2019, demonstrate that the mortality rate of these patients including liver transplantation cases is 11.26% and the liver transplantation rate is 4.35% [41]. Transaminase levels peak around three days after mushroom ingestion, prothrombin time/international normalized ratio worsens during the first three–four days before returning to normal four to seven days after ingestion, while factor V concentrations normalize in about four–five days after mushroom ingestion.

The results from a 12-year retrospective study of 74 patients with amatoxin intoxication in Thailand demonstrate the therapeutic efficacy of N-acetylcysteine [42]. Most patients (70 or 94.59% of the cases) were successfully treated at a low cost. There were four lethal cases. In three patients, death was attributed to late presentation at the hospital. One patient died due to pre-existing advanced alcoholic liver cirrhosis.

Within a prospective case–control study in Assam, India, during the period between April 2014 and April 2015, 94 patients, 57 females and 37 males, with wild mushroom intoxication were assessed [43]. Early hydration therapy, with three to four liters of intravenous fluid per 24 h unless volume overload signs occur along with a continuous nasogastric aspiration, were administered. Pharmacological therapy comprising silimarin, N-acetylcysteine, penicillin G, and vitamin C was additionally administered. There were 13 deaths—eight males (21.62%) and five females (8.77% of the cases).

Through in vivo and in vitro toxicity models, the effects of two antidotes such as resveratrol and silibinin on the prevention of α-amanitin-induced hepatotoxicity were compared [44]. In the first protocol, resveratrol in a dose of 30 mg/kg was given either simultaneously with α-amanitin, or 12 h or 24 h after α-amanitin administration, while silibinin in a dose of 5 mg/kg was given simultaneously either with α-amanitin, or with normal saline. Liver transaminase concentrations after resveratrol and silibinin application were significantly lower than those after the administration of α-amanitin along with normal saline. Resveratrol reduced mononuclear cell infiltration, necrosis, and active caspase-3 immunopositivity in the liver. It was effective in α-amanitin-induced hepatotoxicity associated with *Amanita phalloides* mushroom intoxication, which was attributed to its anti-inflammatory properties under in vivo conditions.

Despite the diversity of pharmacological agents employed in amatoxin poisoning, the accumulated evidence reveals a consistent therapeutic paradigm centered on hepatocellular protection rather than toxin neutralization. The predominance of antioxidant and cytoprotective agents (silibinin, N-acetylcysteine, resveratrol) across different clinical series suggests that the therapeutic strategy has converged on interrupting oxidative cascade pathways rather than directly antagonizing the amatoxin effects. The comparative data from Slovakia, Thailand, and India, while methodologically heterogeneous, consistently demonstrate that combination therapy outperforms monotherapy, and that early initiation is more predictive of outcome than specific agent selection. However, the absence of head-to-head randomized comparisons means that current practice patterns reflect accumulated clinical experience rather than evidence-based optimization of specific drug regimens.

When the currently used pharmacological approaches are compared across the reviewed studies, a consistent pattern emerges despite substantial methodological heterogeneity. The strongest cumulative support can be observed for silibinin and N-acetylcysteine, not because they have been validated in randomized trials, but because they were the only agents that recurred across retrospective cohorts, systematic review data, and contemporary guideline-oriented recommendations. In contrast, penicillin G occupies a transitional position: although still present in several treatment protocols and supported by historical use, its apparent benefit is difficult to separate from the concomitant administration of silibinin, N-acetylcysteine, aggressive hydration, and early supportive care. This makes it difficult to interpret penicillin G as an independently validated intervention. At the opposite end of the evidence spectrum, agents such as corticosteroids, vitamins, rifampicin, etanercept, and other experimental compounds remain hypothesis-generating rather than practice-defining because their support derives primarily from isolated case reports, small cohorts, or preclinical models. Taken together, the available evidence supports a pragmatic best-practice hierarchy in which silibinin and N-acetylcysteine should be regarded as the core pharmacological components of contemporary management, whereas penicillin G may be considered as an adjunctive or alternative option when preferred therapies are unavailable, and experimental agents should be reserved for investigational or highly selected rescue contexts.

Overall, the evidence base remains heterogeneous, with most data derived from retrospective cohorts and systematic reviews of non-randomized studies, limiting the ability to establish definitive treatment hierarchies.

### 5.3. Extracorporeal Detoxification and Blood Purification Techniques

During the period between 2012 and 2019, 11 pediatric patients were diagnosed with pediatric acute liver failure caused by wild mushroom intoxication in the pediatric intensive care unit of the First Hospital of Jilin University, Jilin, China, in a retrospective observational study [45]. The combination of plasma exchange and double plasma molecular adsorption system compensates for the shortcomings of these two management techniques. This was used until liver function improved and coagulation function as normalized in five children, four girls and one boy aged between five and ten years. These patients presented with vomiting and diarrhea. Two patients were categorized as grade IV hepatic encephalopathy. Four patients underwent liver transplantation and one patient died. This therapeutic combination is safe and effective in reducing the bilirubin level and improving blood coagulation in pediatric acute liver failure due to wild mushroom poisoning. Therefore, it can serve as a bridge to transplantation or recovery.

The retrospective investigation of six patients with mushroom intoxication among a total of 59 patients with severe acute toxic hepatitis during the period between 2010 and 2021 in Malatya, Turkey, showed that early therapeutic plasma exchange (within the first 24 h) improved treatment outcomes by reducing circulating toxin concentrations [46].

In a case series of six patients, ingestion of a food preparation later morphologically attributed to *A. fuliginea* was associated with gastrointestinal symptoms, including nausea, vomiting, and diarrhea, followed by hepatic and renal impairment, with a symptom onset latency of 6–12 h [47]. Four of the six patients required hospitalization at Qingdao Hospital of Traditional Chinese Medicine in August 2020. All four hospitalized patients recovered successfully following symptomatic supportive care combined with hemoperfusion or continuous hemodiafiltration.

Two patients with *A. phalloides* intoxication, in which hemodialysis with medium cut-off membrane as a new treatment tool performed in Mugla, Turkey, have been reported [48]. In both cases, there was liver and kidney function improvement. The first patient recovered fully, while the second died despite improvement in renal function due to *Acinetobacter* sepsis.

An isolated case report describes a 56-year-old male patient with severe myocardial damage, multiple organ dysfunction, circulatory failure, recurrent malignant arrhythmia, and cardiac arrest after wild mushroom consumption in Nanchang, China [49]. The patient was treated with venoarterial extracorporeal membrane oxygenation combined with hemoperfusion, plasma exchange, and continuous renal replacement therapy. Their heart rhythm gradually stabilized three hours after the extracorporeal membrane oxygenation surgery and heart function recovered on the sixth day after this procedure. Although clinically informative, this observation is limited by the inherent lack of generalizability of single-case evidence.

The extracorporeal approaches described across these case series and cohort studies illustrate an important strategic evolution: from toxin elimination toward organ support and stabilization. While the theoretical basis for toxin removal remains sound, the clinical reports consistently emphasize improvements in coagulation, bilirubin, and hemodynamic parameters rather than demonstrable toxin clearance. This suggests that the primary value of plasma exchange, DPMAS, hemoperfusion, and specialized dialysis modalities may lie in their capacity to serve as bridge-to-recovery or bridge-to-transplant interventions rather than as definitive detoxification methods. The temporal clustering of successful interventions within the first 24–48 h reinforces the concept that extracorporeal support is most effective when applied as part of an early, aggressive management strategy rather than as salvage therapy for established organ failure.

A comparison of the reviewed extracorporeal modalities also suggests that their reported benefit is less dependent on any single technology than on the clinical context in which they are deployed. Plasma exchange, DPMAS, hemoperfusion, continuous hemofiltration, and newer dialysis configurations are all described in association with improvement in surrogate markers such as bilirubin, coagulation indices, renal function, or hemodynamic stability; however, none are supported by evidence demonstrating clear superiority in patient-centered outcomes. In practical terms, the studies converge on one point more strongly than on any device-specific advantage: extracorporeal support appears most useful when introduced early in deteriorating patients, as part of a broader strategy of stabilization, toxin burden reduction, and bridging to either native recovery or transplantation. Accordingly, current best practice is not to prioritize one extracorporeal modality as universally superior, but to favor the earliest feasible implementation of the most accessible and institutionally supported bridging technique in centers managing severe intoxication.

Although extracorporeal techniques are widely used in clinical practice, their evidence base remains limited and largely derived from small observational studies and case series. Current recommendations consider these methods primarily as supportive or bridging strategies rather than definitive toxin elimination therapies.

### 5.4. Liver Transplantation

Since the mid-1990s, liver transplantation has become a practical option for patients with amatoxin intoxication [38]. This modern method is justified if the prothrombin time is very prolonged and there is evidence of metabolic acidosis, hypoglycemia, and increased serum ammonia. Liver transplantation may be orthotopic or partial.

In a retrospective study including 26 adult patients who underwent emergency liver transplantation for acute liver failure following mushroom intoxication between 2008 and 2023 in Turkey, the overall survival rate was reported to be 69.2%. The analysis demonstrated that higher MELD scores and the need for retransplantation were associated with increased mortality, whereas initial laboratory parameters at hospital admission did not significantly differ between survivors and non-survivors. These findings emphasize the importance of timely referral to specialized transplant centers and the careful selection of candidates for transplantation in patients with severe mushroom poisoning, as early intervention remains a key determinant of clinical outcome [50].

The importance of liver transplantation in the management of severe mushroom poisoning was also confirmed in a systematic review and meta-analysis evaluating 33 studies on mushroom intoxication. The analysis reported mortality rates ranging from 0% to 40%, with a pooled mortality estimate of approximately 2.87%. Among the analyzed cases, 16 patients underwent liver transplantation, of whom 14 survived, further supporting the role of transplantation as a life-saving intervention in patients with fulminant hepatic failure caused by toxic mushrooms. The authors also emphasize that early referral to specialized centers and the timely initiation of appropriate therapy may reduce the need for transplantation and improve clinical outcomes [51].

From a practical triage perspective, transfer to a transplant-capable center should not be delayed until irreversible multiorgan failure has developed. Immediate referral should be strongly considered in any patient with suspected amatoxin-induced acute liver injury who shows rapidly worsening coagulopathy, rising bilirubin, increasing serum creatinine, metabolic acidosis, hepatic encephalopathy, or persistent hyperammonemia despite initial supportive and antidotal therapy. In addition, patients fulfilling or approaching established prognostic thresholds should be transferred early, because the timing of referral may determine transplant eligibility and outcome. Several prognostic systems have been used to guide transplantation decisions in amatoxin poisoning, including Clichy criteria, King’s College criteria, Ganzert criteria, and Escudié criteria. Among these, the Ganzert criteria were specifically developed for amatoxin intoxication and combine a prothrombin index of 25% or less with a serum creatinine concentration of at least 106 μmol/L between days 3 and 10 after ingestion. Escudié criteria, derived from a subsequent reassessment, propose a more stringent threshold—prothrombin index below 10% (approximately INR > 6) from day 4 after ingestion—independent of creatinine, and were found to have 100% accuracy in predicting fatal outcome in their validation cohort. King’s College and Clichy criteria, originally developed for acute liver failure of broader etiologies, require the presence of hepatic encephalopathy as an absolute prerequisite, which limits their applicability in amatoxin poisoning where encephalopathy may develop late or be absent in rapidly fatal cases [52,53,54].

From an analytical standpoint, the reviewed prognostic systems do not appear to have equivalent value in amatoxin poisoning. Criteria developed specifically for this toxicological context, particularly Ganzert and Escudié criteria, are conceptually more aligned with the clinical trajectory of amatoxin-induced liver injury because they emphasize evolving coagulopathy and renal dysfunction rather than requiring overt encephalopathy as a prerequisite. In contrast, broader acute liver failure criteria such as King’s College and Clichy may remain useful as general reference frameworks, but they are less well adapted to the distinctive timing of amatoxin toxicity, in which critical deterioration may precede classical encephalopathic thresholds. Accordingly, current best practice would favor the early use of amatoxin-specific prognostic thinking, combined with serial dynamic reassessment, rather than delayed reliance on end-stage criteria originally designed for other causes of acute liver failure.

### 5.5. Emerging and Experimental Therapeutic Approaches

The retrospective investigation of 61 adult patients with acute mushroom poisoning and liver injury hospitalized in seven hospitals in China from May 2016 to May 2021 demonstrates that in the group treated with the medicinal mushroom *Ganoderma lucidum*, length of hospital stay was statistically significantly shorter than in the control group (6.69 ± 3.98 days vs. 9.27 ± 5.30 days; t = 2.174; *p* = 0.034) [55]. Furthermore, there were statistically significantly lower hospitalization expenses in the first than in the second group (16,336.49 ± 12,615.76 Chinese yuan vs. 27,540.08 ± 23,709.57 Chinese yuan; t = 2.382; *p* = 0.020). There were statistically significantly fewer patients with a blood purification treatment time >48 h in the first than in the second group (30% vs. 69.23%; χ^2^ = 4.891; *p* = 0.027).

Two patients with acute *A. phalloides* intoxication following mushroom ingestion who were successfully treated with intravenous rifampicin in Heidelberg, Australia, have been reported [56].

Two cases of mushroom α-amanitin-induced liver injury presenting with gastrointestinal disorder on admission in Shanghai, China, have been reported [57]. Their treatment was accomplished under the close monitoring of laboratory examinations and included the off-label medication of etanercept, a tumor necrosis factor-α blocker. Tumor necrosis factor-α plays an important role in liver injury aggravation and initiation of the inflammatory cascade. Although therapy with its blockers possesses potential therapeutic efficacy in hepatic dysfunction, its safety in liver injury still lacks strong evidence.

These reports illustrate the heterogeneous and exploratory nature of emerging therapeutic strategies in severe mushroom poisoning. While agents such as *Ganoderma lucidum*, rifampicin, and tumor necrosis factor-α inhibitors demonstrate potential clinical benefits, ranging from reduced hospitalization time to possible hepatoprotective effects, the available evidence remains limited to small retrospective analyses and isolated case reports. Importantly, these interventions appear to target different aspects of the pathophysiological cascade, including toxin metabolism, inflammatory signaling, and oxidative stress, suggesting that future therapeutic approaches may need to adopt a multimodal strategy rather than rely on a single agent. The absence of controlled clinical trials and the variability in study design significantly limit the generalizability of these findings, and these therapies should therefore be regarded as investigational rather than established treatment options.

### 5.6. Selected Syndrome-Specific Cases Illustrating Diagnostic and Therapeutic Heterogeneity

Because therapeutic decision-making in mushroom poisoning cannot be reduced entirely to amatoxin-related liver injury, selected case-based reports from other toxic syndromes are included here to illustrate clinically important variations in presentation, organ involvement, and supportive management priorities.

A 64-year-old man with substernal chest discomfort, nausea, vomiting and myalgia lasting for 12 h, and elevated serum high-sensitive troponin I was initially misdiagnosed with non-ST segment elevation myocardial infarction, while two other family members having also consumed *Russula subnigricans* were diagnosed with mushroom poisoning complicated by severe rhabdomyolysis in Yangsan, Korea [58]. After admission, successful conservative treatment with primary fluid resuscitation was carried out and the patients were discharged without any complications. This case is clinically instructive because it demonstrates that not all severe mushroom intoxications present primarily with hepatotoxicity; in some syndromes, early recognition depends on identifying atypical patterns such as rhabdomyolysis and myocardial biomarker elevation, which may initially mimic non-toxicological emergencies.

One patient with *A. neoovoidea* intoxication in Chuxiong, China has been reported [59]. The patient presented with nausea, vomiting, oliguria, and acute renal function injury. He was discharged after symptomatic support treatment and blood purification therapy. The relevance of this report lies in underscoring that severe mushroom poisoning may also manifest predominantly as acute kidney injury, further supporting the need for diagnostic and therapeutic frameworks that remain syndrome-aware rather than exclusively hepatocentric.

Overall, the contemporary management of wild mushroom intoxications is based on a multimodal therapeutic strategy integrating supportive care, pharmacological antidote therapy, extracorporeal detoxification techniques, and in severe cases, liver transplantation. Early recognition of the toxic syndrome and timely escalation of care remain the cornerstones of management (Table 2). Pharmacological therapies such as silibinin, penicillin G, and N-acetylcysteine aim to limit hepatocellular injury and oxidative stress, while extracorporeal purification techniques may reduce circulating toxin levels and stabilize critically ill patients. In cases of fulminant hepatic failure, liver transplantation remains the only definitive life-saving intervention. At the same time, emerging therapeutic approaches and experimental pharmacological agents continue to be investigated in order to improve survival and reduce the need for transplantation. Taken together, these advances highlight the importance of early diagnosis, multidisciplinary management, and timely referral to specialized centers in the treatment of severe mushroom poisoning.

These syndrome-specific reports also expose an important limitation of predominantly hepatotoxic management frameworks: they risk delaying recognition in patients whose initial presentation is dominated by rhabdomyolysis, myocardial injury, or acute kidney injury rather than liver dysfunction. A comparison between these non-amatoxin presentations and the classic amatoxin model suggests that early management should be guided less by an assumed toxin category and more by dynamic syndrome recognition, organ-specific monitoring, and escalation triggers tailored to the dominant pattern of injury. In practical terms, best practice in emergency settings should therefore remain syndrome-aware rather than exclusively hepatocentric, particularly when the exposure history is uncertain and the presenting phenotype initially mimics non-toxicological disease.

From a practical clinical perspective, management of suspected mushroom poisoning should be stratified according to the time elapsed since ingestion, severity of clinical presentation, and availability of specialized resources. In patients presenting within the first 24 h, including those in the asymptomatic latent phase, priority should be given to gastrointestinal decontamination with activated charcoal and the prompt initiation of antidotal therapy with intravenous silibinin and/or N-acetylcysteine, as the efficacy of both decontamination and pharmacological intervention is critically time-dependent. In cases with evolving laboratory abnormalities or evidence of hepatic or renal involvement, management should escalate to intensive monitoring, aggressive supportive care, and the consideration of extracorporeal support techniques, including plasma exchange or double plasma molecular adsorption system (DPMAS), in centers with appropriate capabilities. Patients demonstrating signs of acute liver failure, including worsening coagulopathy, rising bilirubin, encephalopathy, metabolic acidosis, or fulfillment of established prognostic thresholds such as the Escudié criteria or Ganzert criteria, require urgent transfer to transplant-capable centers, as the timing of referral directly determines transplant eligibility and outcome [50,51,52,53,54]. In resource-limited settings where access to advanced extracorporeal therapies or liver transplantation is restricted, emphasis should be placed on the early recognition of the toxic syndrome, aggressive supportive care, and timely referral to higher-level facilities when feasible. In the absence of randomized controlled trial data, this stratified framework represents the current best-practice synthesis derived from retrospective cohort evidence, expert consensus, and applicable guideline recommendations [36,37].

## 6. Discussion

### 6.1. Heterogeneity of Evidence and Study Design Limitations

A central limitation of the available literature is the marked heterogeneity in study design and evidence quality. The current evidence base is dominated by retrospective cohorts, small observational studies, and case reports, with very limited prospective or controlled data. As a result, many conclusions are based on associative findings and clinical experience rather than robust causal inference, limiting the strength of the therapeutic and prognostic recommendations [1,2,14,38].

A parallel observation concerns the structural shift in the diagnostic paradigm. Classical symptom-based assessment remains clinically indispensable but is increasingly insufficient as a standalone approach. This reflects the unreliability of anamnesis and the time-critical nature of therapeutic decisions. The integration of analytical toxicology, molecular identification, and computational triage tools reflects a transition toward a precision medicine framework in emergency toxicology. However, this approach remains constrained by limited external validity and the predominance of single-center data. This limitation is not unique to mushroom toxicology but reflects a broader challenge in rare, high-severity toxic exposures where randomized study designs are often not feasible.

### 6.2. Latency Period and Clinical Syndrome Classification

The latency interval between mushroom ingestion and symptom onset remains a key initial clinical parameter, but its prognostic interpretation requires caution. Prolonged latency (>6 h) is more consistently associated with hepatotoxic or nephrotoxic syndromes, whereas short-latency presentations are not inherently benign. Severe muscarinic toxidrome caused by *Clitocybe* or *Inocybe* species may develop within 30 min and can be life-threatening due to bronchospasm and cardiovascular compromise, particularly in elderly patients and children. Early gastrointestinal symptoms may mask the latent phase of a long-latency syndrome, leading to the underestimation of exposure severity. This temporal pattern has a clear toxicological basis. Amatoxins inhibit RNA polymerase II, blocking protein synthesis in hepatocytes, so clinical manifestations occur only after significant cellular injury has developed. Accordingly, early symptom onset does not necessarily indicate greater severity, whereas prolonged latency is more often associated with severe organ damage [1,2,5,7,16]. Latency should therefore be regarded as an orienting rather than definitive prognostic marker and interpreted within a toxin-specific pathophysiological framework. Importantly, the absence of early symptoms does not exclude significant toxin burden and should not delay treatment.

The classic four-phase model of intoxication continues to provide a useful conceptual framework for understanding the clinical course [8,14]. A clinically important feature is the phase of apparent recovery, during which gastrointestinal symptoms temporarily subside despite ongoing hepatic and renal injury. Available clinical observations indicate that this stage is one of the main diagnostic pitfalls, leading to misinterpretation of the condition as uncomplicated gastroenteritis and premature discharge of the patient. Accordingly, serial monitoring of liver enzymes, coagulation parameters, and ammonia should be regarded as an integral component of early diagnostic assessment. This diagnostic pitfall is not merely academic: premature discharge during the apparent recovery phase has been documented as a contributing factor in preventable fatalities.

### 6.3. Advances in Analytical Toxicology and Molecular Identification

A review of modern laboratory methods shows a trend toward increasing analytical specificity in the detection of toxic compounds. Methods such as liquid chromatography coupled with mass spectrometry, as well as various immunoanalytical techniques, allow for the direct detection of toxins such as α-, β- and γ-amanitin in biological samples [10,11,12]. However, the available data also highlight several important limitations. Most of these methods require highly specialized equipment, expert personnel and significant financial resources, which limits their accessibility in many clinical centers. Therefore, despite their high analytical sensitivity, their application in routine emergency practice remains limited.

Molecular genetic approaches, including PCR-based assays, loop-mediated isothermal amplification, and MALDI-TOF mass spectrometry, offer a qualitatively different capability: species-level identification from degraded biological material, including gastric contents or food remnants, under conditions where morphological assessment is impossible. This is of particular clinical relevance in cases of late presentation or anonymous exposure, where the toxic species cannot be established by conventional means. However, the current evidence base for these methods consists predominantly of analytical validation studies rather than prospective clinical outcome data, and their role in influencing real-time management decisions remains to be formally established [20,21,22,23].

Analytical capability should therefore be distinguished from clinical applicability. Although molecular and mass spectrometric methods perform well under controlled conditions, their emergency use is limited by logistics, technical requirements, and turnaround time. Their current role is therefore mainly complementary. In clinical practice, the greatest value lies in tools that provide actionable information within the therapeutic window for referral, decontamination, antidotal treatment, and escalation of care. Rapid toxin-oriented assays and integrated prognostic models may therefore be more immediately useful than highly sophisticated methods with delayed availability [1,38].

### 6.4. Digital Technologies, Artificial Intelligence, and Public Health Implications

The growing incorporation of digital technologies and artificial intelligence into mushroom toxicology also warrants careful cautionary framing. Early machine learning models show promising results in severity assessment and outcome prediction. However, their performance depends on dataset representativeness and requires external validation across different geographic and clinical settings. A similar, and in some respects more concerning, limitation applies to smartphone applications for visual mushroom recognition, whose accuracy remains insufficient for safe real-world use. Beyond their imperfect diagnostic performance, these tools may create false reassurance and thereby contribute to delayed consultation, inappropriate self-triage, or unsafe mushroom consumption. For this reason, they should be regarded only as auxiliary or educational tools and not as instruments for determining edibility or excluding toxic exposure. This position is aligned with recent public health warnings in Europe; for example, ANSES has explicitly advised against consuming mushrooms identified solely by smartphone recognition applications because of the high risk of misidentification [60].

### 6.5. Therapeutic Landscape: Evidence Gradients and Current Practice

The treatment of wild mushroom poisoning is increasingly defined by timing rather than by pharmacological certainty. A critical window exists between nonspecific symptoms and the development of severe organ damage, during which early intervention strongly influences prognosis. Accordingly, current management has shifted from a reactive model toward early risk stratification, aggressive supportive care, and timely escalation. Extracorporeal therapies are most effective when applied before irreversible cellular injury occurs, underscoring the importance of early recognition and referral.

A defining feature of the current pharmacological evidence base is the discrepancy between widespread clinical use and limited methodological robustness. Silibinin and N-acetylcysteine are consistently incorporated into treatment regimens, yet their evidence derives primarily from retrospective cohorts and non-randomized analyses. Their clinical consistency across different series likely reflects a broader pathophysiological role, encompassing antioxidant, cytoprotective, and anti-inflammatory mechanisms rather than a single targeted effect. Penicillin G, historically considered a cornerstone of amatoxin management, is supported by mechanistically plausible but clinically inconsistent data, and its independent contribution remains unclear. Emerging agents such as rifampicin, resveratrol, and etanercept target different elements of the pathophysiological cascade but remain clinically unvalidated. This is reflected in the current guidelines, which assign only conditional recommendations with very low-quality evidence, highlighting a treatment paradigm driven by clinical necessity rather than controlled data [37].

Despite these limitations, the available evidence is sufficiently consistent to support a pragmatic clinical approach. In suspected severe amatoxin poisoning, best practice relies on early implementation of a multimodal strategy rather than on a single intervention. This includes decontamination, supportive care, silibinin and/or N-acetylcysteine, dynamic monitoring, and early referral to specialized centers. Within this framework, pharmacological agents form the therapeutic backbone, while extracorporeal techniques and transplantation serve as escalation strategies guided by disease progression. Thus, the most defensible approach is not the selection of a single best therapy, but the early combination of available interventions before irreversible organ failure develops.

### 6.6. Extracorporeal Support and Liver Transplantation

The review of extracorporeal methods also supports the idea that the therapeutic management of severe fungal intoxications is becoming increasingly complex and resource-intensive. Plasma exchange, DPMAS, hemoperfusion, continuous hemofiltration, and newer dialysis variants should be viewed not merely as technical alternatives, but as components of bridging strategies aimed at stabilization until recovery or transplantation. Despite promising clinical observations, the evidence supporting extracorporeal techniques remains limited in scope and methodological rigor, precluding definitive conclusions regarding the comparative effectiveness of specific modalities. Nevertheless, the consistent observation that early application of these methods is associated with improvements in coagulation, bilirubin, and overall clinical stability suggests that they have the greatest value as part of a broader “bridge-to-recovery” or “bridge-to-transplant” strategy.

Liver transplantation occupies a distinct role in current management, representing the transition from supportive therapy to definitive life-saving intervention in selected patients with fulminant liver failure. The available evidence indicates that outcome depends less on the mere availability of transplantation than on timely referral to a transplant-capable center. Higher MELD scores and the need for retransplantation are associated with increased mortality, while initial laboratory parameters are often insufficient to distinguish survivors from non-survivors. This emphasizes the need for dynamic prognostic assessment rather than delayed reliance on static admission values.

From a practical standpoint, referral should not await overt encephalopathy or multiorgan failure. Several prognostic criteria have been proposed for amatoxin-induced acute liver failure. Ganzert criteria define a threshold of prothrombin index ≤ 25% combined with serum creatinine ≥106 μmol/L between days 3 and 10 after ingestion, but their accuracy is limited because renal impairment may be absent even in patients requiring transplantation. Escudié criteria apply a more stringent threshold, namely prothrombin index <10% (approximately INR > 6) from day 4 onward, without requiring encephalopathy, and have demonstrated high accuracy in validation cohorts. However, fulfilment of the Escudié criteria leaves a mean interval of only approximately 29 h until death or transplantation, which may be insufficient to identify a compatible donor. King’s College and Clichy criteria, originally developed for broader etiologies of acute liver failure, require encephalopathy as an absolute prerequisite, limiting their applicability in amatoxin poisoning where encephalopathy may be absent or appear late. In practice, referral decisions should be guided by the dynamic trajectory of coagulopathy and hepatic dysfunction rather than by single-threshold fulfilment. Transfer should be initiated as soon as progressive coagulation impairment becomes evident, ideally before established prognostic criteria have been met [50,51,61].

### 6.7. Emerging and Experimental Therapies

The growing interest in experimental and emerging therapies, such as *Ganoderma lucidum*, rifampicin, and TNF-α blockade with etanercept, demonstrates that the field is actively seeking solutions beyond conventional regimens. These agents act at different points within the pathophysiological cascade, including hepatocellular protection, interference with toxin uptake, and modulation of inflammatory signaling. This mechanistic diversity suggests that future therapeutic progress may depend more on rational multimodal combinations than on any single investigational agent. At present, however, this concept remains hypothetical, as the available evidence is limited to small retrospective observations, isolated case reports, and early experimental data, without validation in controlled clinical studies [55,56,57].

### 6.8. Syndromic Heterogeneity Beyond Amatoxin Poisoning

The reviewed cases expand the clinical spectrum of mushroom intoxication beyond the classic hepatorenal model. Presentations such as rhabdomyolysis, myocardial injury, or acute kidney injury may mimic other emergencies and delay toxicological recognition. This has important implications for emergency care, as the clinical risk extends beyond the underrecognition of hepatotoxic syndromes to include delayed identification of severe non-hepatocentric presentations. From a systems perspective, this highlights a limitation of the current triage pathways, which remain largely anchored to hepatotoxic patterns. Patients presenting with rhabdomyolysis, arrhythmia, or acute kidney injury may not trigger the same level of urgent referral as those with overt hepatic injury, despite comparable severity [58,59].

Although the present review is centered primarily on amatoxin intoxication, the selective inclusion of other mushroom poisoning syndromes was methodologically intentional. These studies were used not to suggest an equivalent depth of evidence across all syndromes, but to highlight the marked heterogeneity of clinical presentation, organ tropism, and diagnostic pathways in mushroom toxicology. They also illustrate that several emerging analytical and molecular approaches may have value beyond a single toxin class, while at the same time underscoring the limitations of applying amatoxin-centered severity models uniformly across all toxic mushroom exposures.

### 6.9. Research Gaps

A critical analysis of the literature reveals several research gaps. High-quality prospective and multicenter studies directly comparing therapeutic strategies are lacking. Heterogeneity in study populations and toxin profiles complicates the interpretation of outcomes, and no universally accepted criteria currently define the optimal timing for therapeutic escalation or transplantation referral. The main unresolved questions are now comparative and operational: which patients benefit most from extracorporeal support, whether combination therapy is superior, and which thresholds should trigger early transfer. The current literature is rich in proof-of-concept observations but still poor in comparative clinical architecture, which is precisely the level of evidence needed to move from accumulated experience to standardized algorithms.

An additional limitation relates to the geographic distribution of the available evidence. A large proportion of clinical studies and case series originate from regions such as China, Thailand, India, and Turkey, where both the spectrum of toxic mushroom species and the organization of healthcare systems differ from those in Western Europe and North America [17,42,43,50]. These differences may influence exposure patterns, diagnostic approaches, and treatment strategies, and should be considered when extrapolating findings across different clinical and epidemiological settings. In China and Southeast Asia, the predominant amatoxin-producing species include *A. exitialis* and *A. subjunquillea*, which differ from the European *A. phalloides* in regional distribution, and to some extent, in toxin composition, potentially influencing the clinical course and outcome data reported in these studies. At the same time, the high case volume in these regions provides valuable clinical insight into severe intoxication patterns that may be less frequently encountered elsewhere.

In conclusion, progress in the treatment of wild mushroom intoxications is determined not by the emergence of a universal antidote, but by the improvement of early recognition, more precise risk stratification, multimodal treatment approaches, and timely referral to specialized centers. Future progress in this field will depend on the integration of pathophysiological knowledge with high-quality clinical research that transforms accumulated empirical experience into standardized, evidence-based therapeutic algorithms.

## 7. Conclusions

The analysis of the available literature strongly suggests that wild mushroom poisonings can no longer be considered as isolated toxicological incidents, assessed solely by clinical symptoms or the identified mushroom species. The accumulated data outline a clear shift toward an integrative model in which the outcome of intoxication is determined by the interaction between time to recognition, the accuracy of early risk stratification, the dynamics of organ damage, and the timely inclusion of escalating therapeutic interventions. Overall, clinical outcome appears to depend not only on toxin type, but also on timely recognition, accurate risk stratification, and coordinated escalation of care.

At the same time, the review reveals significant gaps in knowledge. Despite advances in toxicological verification, molecular identification, and extracorporeal detoxification methods, the evidence base remains fragmented, predominantly retrospective, and methodologically heterogeneous. Prospective multicenter studies are lacking to validate uniform prognostic thresholds, directly compare pharmacological regimens, and determine the optimal time window for implementing detoxification and transplantation strategies. It also remains unclear which biomarkers, clinical scores, and dynamic laboratory profiles should trigger early referral to a transplant center.

Taken together, the available evidence supports a time-sensitive, multi-level, and syndrome-oriented framework for clinical management. In this framework, diagnosis is not merely sequential to therapy. Rather, it actively informs the timing, intensity, and escalation of treatment. Risk should be reassessed dynamically, and management should proceed as a stepwise escalating strategy involving early clinical recognition, toxicological and molecular confirmation, sequential assessment of severity, early hepato- and organoprotective intervention, timely application of extracorporeal support, and early, rather than delayed, transplantation thinking.

Therefore, future development of the field should not be directed toward the search for a universal antidote, but toward the construction of standardized, validated, and interoperable clinical algorithms that connect pathophysiology, analytical toxicology, and intensive care medicine into a single decision-making path. Such an approach may improve the consistency, timeliness, and clinical effectiveness of management in severe mushroom intoxication.

## Figures and Tables

**Table 1:** Summary of studies on modern diagnostic methods for wild mushroom intoxications.

Author(s), Year	Country/Region	Study Objective	Study Design	Level of Evidence	Population/Sample	Main Findings	Reference
Kieslichová, 2021	Czech Republic	To outline the basis for diagnosis of *A. phalloides* poisoning	Narrative review	Low	Not specified	Diagnosis relies on ingestion history, gastrointestinal symptoms, typical time course, laboratory markers, and mycological or toxicological examination	[16]
Dluholucký et al., 2022	Slovakia	To quantitatively determine amanitins in blood and urine of patients with suspected *A. phalloides* poisoning using ELISA	Prospective analytical cohort study	Moderate–High	698 patients with suspected *A. phalloides* poisoning	Urinary amanitin correlated with poisoning severity (6–47 h post-ingestion) with no false negatives; serum amanitin had no diagnostic value	[17]
Liu et al., 2023	China	To construct an early triage model using machine learning for critically ill mushroom poisoning patients	Retrospective ML model development and validation study	Moderate	567 critically ill adult mushroom poisoning patients; training (n = 322) and test (n = 245) cohorts	XGBoost showed best performance: AUC 0.83 (CV) and 0.90 (test); sensitivity 0.93, specificity 0.79; outperformed physicians (sensitivity 0.86, specificity 0.66)	[18]
Ye et al., 2021	China	To evaluate laboratory markers and clinical scoring systems for mortality prediction in *A. phalloides* poisoning	Retrospective cohort study (2009–2018)	Moderate	105 patients with *A. phalloides* intoxication from two university hospitals	INR > 3.6 (AUC 0.941) and plasma ammonia > 95.1 μmol/L (AUC 0.805) were independently associated with mortality; CLIF-OF score > 9 at 24 h achieved >90% diagnostic accuracy, outperforming other scoring systems	[19]
Khatir et al., 2020	Iran	To identify statistically abnormal laboratory parameters in hospitalized wild mushroom intoxication patients	Descriptive retrospective cross-sectional study (4-year period)	Low–Moderate	65 hospitalized patients (Razi Hospital, Mazandaran)	ALT, INR, PT, and aPTT were statistically significantly abnormal (*p* = 0.003, 0.006, 0.035, 0.050, respectively)	[20]
Yan et al., 2023	Not stated	To review analytical tools and data analysis methods for identification and quality evaluation of wild edible mushrooms	Systematic narrative review (>167 articles, 20 years)	Moderate	Wild edible mushroom species literature	Five macro/microscopic/molecular identification techniques reviewed; chromatography and spectroscopy combined with chemometrics applied for quality evaluation; deep learning showed advantages in image recognition	[21]
Gao et al., 2022	China	To develop rapid and sensitive methods for detecting *A*. *citrinoannulata*	Method development and validation study (colorimetric and real-time LAMP)	Low	41 non-target mushroom species; fresh, cooked, and vomit samples	Both assays detected 0.2 ng *A. citrinoannulata* DNA without cross-reactions; assay completed in 40 min; visible results; 1% target content detectable in mixed samples	[22]
He et al., 2019	Not stated	To detect and distinguish different lethal *Amanita* species using LAMP and HRCA	Methodological comparative study	Low–Moderate	10 lethal *Amanita* spp. (section *Phalloideae*); 16 non-*Phalloideae Amanita* spp.	LAMP discriminated introclade but not intraclade species; HRCA discriminated both; detection limits: 10 pg (LAMP) and 1 pg (HRCA) genomic DNA per reaction	[23]
Xie et al., 2022	Not stated	To develop a visual, rapid, and cost-effective LAMP method for *Gyromitra infula* identification	Method development and validation study	Low	Boiled and gastric juice-digested mushroom samples; mixtures containing target species	Minimum detectable DNA: 1 ng/μL; 1% *G. infula* content detected in processed samples; assay completed within 90 min with naked-eye visible results	[24]
Wang et al., 2022	Not stated	To develop real-time fluorescence and visual LAMP assays for *Russula senecis* detection	Method development, optimization, and validation study	Low	Fried and digested mushroom samples; mushroom mixtures containing target species	Detection limit: 3.2 pg genomic DNA; 1% target species in mixtures reliably identified; visual system optimized to minimize reaction time	[25]
Piarroux et al., 2021	France	To create and internally validate a MALDI-TOF MS reference database for common *Amanita* species	Reference database development and internal validation study	Low-Moderate	15 *Amanita* species; 38 field specimens from four French regions	Database successfully validated for 15 species; decayed *A. phalloides* portions correctly identified by MALDI-TOF MS via free online spectral matching application	[26]
Sugano et al., 2022	Japan	To develop a quick and specific LAMP method for detecting *Omphalotus japonicus*	Method development and validation study	Low	13 edible mushroom species (cross-reactivity testing); mixed mushroom samples	Amplification within 60 min; full detection (including DNA extraction) within 2 h; no cross-reactivity with 13 edible species; 1% target detectable in mixed samples	[27]
Zhang et al., 2021	Not stated	To design multilocus PCR-HRM primers for identification of *Psilocybe cubensis* DNA	Method development study	Low	*Psilocybe cubensis* and comparison mushroom species	Four target markers (RPB1, PPT, GAPDH, EF1α) with distinct melting temperatures established; significant HRM signal at 62.5 pg/μL; rapid and specific species differentiation achieved	[28]
Parnmen et al., 2019	Thailand	To identify *Cantharocybe virosa* as the causative agent in a gastrointestinal poisoning outbreak	Case-linked outbreak investigation using molecular and mass spectrometric analyses	Moderate	39 poisoning patients in Thailand	*C. virosa* identified by ITS and LSU rDNA sequence analyses and confirmed by LC-QTOF-MS; species linked to gastrointestinal syndrome	[29]
Zhu et al., 2021	Not stated	To establish an automated magnetic bead-based chemiluminescence immunoassay for early diagnosis of wild mushroom poisoning	Analytical method development and validation study	Low	Human serum and urine samples	LOD: 0.010 ng/mL (serum) and 0.009 ng/mL (urine); recoveries 81.6–95.6%; CV <12.9%; fully automated using integrated device	[30]
Bambauer et al., 2021	Not stated	To reduce analysis time by targeting biomarkers of late- and early-onset toxic mushroom syndromes in urine using HILIC-HRMS	Method development and applicability study	Low	10 urine samples from patients with suspected wild mushroom poisoning	Two validated urine methods: (i) ricinine, α- and β-amanitin; (ii) muscarine, muscimol, ibotenic acid; α- and β-amanitin, muscarine, muscimol, and ibotenic acid identified; psilocin-O-glucuronide distinguished from bufotenine-O-glucuronide in two samples	[31]
Abbott et al., 2018	Not stated	To develop a validated LC-MS/MS method for detecting α-, β-, and γ-amanitin in urine	Analytical method development and validation study	Low	Pooled human urine samples	α-Amanitin: precision ≤ 5.49%, accuracy 100–106%, range 1–200 ng/mL; β- and γ-amanitin: precision ≤ 17.2%, accuracy 99–105%; calibration ranges 2.5–200 and 1.0–200 ng/mL, respectively	[32]
Yoshioka et al., 2020	Japan	To quantify ustalic acid in *Tricholoma ustale* by LC-MS/MS after solid-phase extraction	Analytical quantification method development study	Low	Shiitake mushroom, miso soup, and leftover food poisoning case samples	LOQ: 10 ng/g (mushroom) and 0.40 ng/g (miso soup); accuracy 99.8–105% (mushroom) and 98.8–102% (miso); ustalic acid detected at 0.57–3.7 μg/g in case samples	[33]
Liu et al., 2023a	Not stated	To identify serum metabolic alterations and diagnostic biomarkers in amatoxin poisoning using untargeted metabolomics	Case–control study using UHPLC-QTOF-MS/MS	Moderate	61 amatoxin poisoning patients; 61 matched healthy controls	33 differential metabolites (15 up-regulated, 18 down-regulated); pathways: glycerophospholipid, sphingolipid, amino acid metabolism; 8 metabolic markers with AUC > 0.8; 11-oxo-androsterone glucuronide, glucose 6-phosphate, and glycochenodeoxycholate-3-sulfate positively correlated with liver injury	[34]
Hodgson et al., 2023	Australia (Melbourne)	To compare the accuracy of three smartphone mushroom identification applications	Comparative accuracy study using digital photographs	Low	78 mushroom specimens photographed (2020–2021)	Overall accuracy: Picture Mushroom 49%, Mushroom Identificator 35%, iNaturalist 35%; poisonous species: 44%, 30%, 40%; *A. phalloides*: Mushroom Identificator 67%, Picture Mushroom 60%, iNaturalist 27%	[35]

**Table 2:** Summary of studies on advances in the treatment of wild mushroom intoxications.

Author(s), Year	Country/ Region	Study Objective	Study Design	Level of Evidence	Population/ Sample	Intervention/Main Findings	Clinical Relevance	Reference
Kieslichová, 2021	Czech Republic	To outline the current treatment framework for *A. phalloides* intoxication	Narrative review	Low	Not specified	Treatment includes detoxification procedures, supportive care, pharmacological agents, and ICU management; urgent liver transplantation is the only life-saving option in selected patients with acute liver failure	Establishes the foundational treatment framework guiding the clinical management of amatoxin poisoning	[16]
Vetter, 2023	Not stated	To review therapeutic approaches for amatoxin poisoning from symptom onset to advanced interventions	Narrative review	Low	Not specified	Therapy includes fluid/electrolyte replacement, activated charcoal, hemodialysis, hemoperfusion, plasmapheresis, MARS, and chemotherapy with natural/synthetic molecules; early initiation is critical	Provides a comprehensive overview of the sequential therapeutic strategy for amatoxin poisoning from initial to advanced stages	[38]
Wennig et al., 2020	Not stated	To summarize the four main therapeutic options for amatoxin intoxication	Narrative review	Low	Not specified	Four treatment pillars: (i) volume replacement with electrolytes; (ii) toxin binding/elimination (hemodialysis, activated charcoal); (iii) antidote therapy (penicillin G, silibinin, N-acetylcysteine); (iv) liver failure management including transplantation	Concise classification of treatment modalities widely used as a clinical reference framework	[1]
Xue et al., 2023	Not stated	To highlight the current absence of specific antidotes for α-amanitin and the reliance on symptomatic therapy	Narrative review/commentary	Low	Not specified	No specific detoxification drug exists for α-amanitin; clinical management currently relies entirely on symptomatic and supportive therapy	Underscores the unmet therapeutic need for targeted antidotes and motivates ongoing pharmacological research	[39]
Le Daré et al., 2021	Not stated	To review the role of antioxidant antidotes and their mechanisms of action in *A. phalloides* poisoning	Narrative review	Low	Not specified	Antidotes with antioxidant properties are the most effective therapeutics; oxidative stress plays a predominant pathophysiological role; partially elucidated mechanisms suggest potential targets for new antidote development	Supports antioxidant-based pharmacological strategies and identifies mechanistic targets for novel antidote development	[40]
Sezer & Ilhan, 2021	Not stated	To summarize drugs applied in the management of *A. phalloides* intoxication	Narrative review	Low	Not specified	Multiple agents used alone or in combinations: penicillin G, silibinin, N-acetylcysteine, thioctic acid, corticosteroids, ceftazidime, cimetidine, vitamins C and E, insulin, glucagon, and human growth hormone	Provides a comprehensive overview of pharmacological agents employed in clinical practice for amatoxin poisoning	[14]
Dluholucký et al., 2022	Slovakia	To compare outcomes of combined penicillin G + silibinin vs. silibinin monotherapy in confirmed *A. phalloides* intoxication	Retrospective comparative cohort study (2004–2020)	Moderate	141 patients: 129 treated with penicillin G + silibinin; 12 with silibinin only	Combination therapy: 2 deaths (acute kidney injury); monotherapy: 4 deaths (fulminant liver failure, intracranial hemorrhage), 1 liver transplantation; treatment failure significantly higher with monotherapy (41.67% vs. 1.57%; *p* = 0.00058)	Strongly supports combined penicillin G and silibinin as the superior antidote regimen over silibinin monotherapy	[17]
Liu et al., 2020	Not stated (multi-DB)	To assess the efficacy of N-acetylcysteine treatment in amatoxin poisoning through systematic review	Systematic review (PubMed, EMBASE, CENTRAL, SinoMed; up to August 2019)	Moderate–High	13 studies; 506 patients with amatoxin poisoning treated with N-acetylcysteine	Mortality rate (including transplantation): 11.26%; liver transplantation rate: 4.35%; transaminases peaked ~day 3; PT/INR normalized by day 4–7; factor V normalized by day 4–5	Provides the most robust evidence for N-acetylcysteine efficacy and characterizes the temporal evolution of hepatotoxic markers	[41]
Jongthun et al., 2022	Thailand	To evaluate the therapeutic efficacy of N-acetylcysteine over 12 years of clinical practice	Retrospective cohort study (12-year period)	Moderate	74 patients with amatoxin intoxication	70 patients (94.59%) successfully treated at low cost; 4 deaths: 3 due to late hospital presentation, 1 due to advanced alcoholic liver cirrhosis	Confirms N-acetylcysteine as a cost-effective therapeutic option with high survival rates when treatment is initiated early	[42]
Dutta et al., 2018	India (Assam)	To evaluate outcomes of a multi-drug protocol including silimarin, N-acetylcysteine, penicillin G, and vitamin C in wild mushroom poisoning	Prospective case–control study (April 2014–April 2015)	Moderate–High	94 patients (57 females, 37 males) with wild mushroom intoxication	Early IV hydration (3–4 L/24 h), nasogastric aspiration, silimarin, N-acetylcysteine, penicillin G, and vitamin C administered; 13 deaths: 8 males (21.62%), 5 females (8.77%)	Highlights the higher mortality risk in male patients and supports early multimodal pharmacological intervention	[43]
Sahin et al., 2018	Not stated	To compare the hepatoprotective effects of resveratrol and silibinin against α-amanitin-induced hepatotoxicity	Experimental in vivo and in vitro study	Low	Animal and cell culture toxicity models	Resveratrol (30 mg/kg) and silibinin (5 mg/kg) significantly reduced liver transaminases vs. α-amanitin alone; resveratrol reduced mononuclear infiltration, necrosis, and caspase-3 immunopositivity through anti-inflammatory mechanisms	Supports resveratrol as a promising anti-inflammatory antidote candidate for α-amanitin hepatotoxicity, complementing silibinin	[44]
Yang et al., 2021	China (Jilin)	To evaluate the combined use of plasma exchange and DPMAS in pediatric acute liver failure caused by wild mushroom poisoning	Retrospective observational study (2012–2019)	Moderate	11 pediatric patients with acute liver failure (Pediatric ICU, First Hospital of Jilin University)	Combination of plasma exchange and DPMAS used until liver and coagulation function normalized in 5 patients; 4 underwent liver transplantation; 1 death; combination reduced bilirubin and improved coagulation	Supports combined plasma exchange and DPMAS as a safe and effective bridge to transplantation or recovery in pediatric patients	[45]
Berber et al., 2021	Turkey (Malatya)	To assess the impact of early therapeutic plasma exchange on outcomes in mushroom-related toxic hepatitis	Retrospective cohort study (2010–2021)	Moderate	6 mushroom poisoning patients among 59 with severe acute toxic hepatitis	Early plasma exchange (within first 24 h) reduced harmful substance concentrations and improved treatment outcomes	Supports early initiation of therapeutic plasma exchange as a key determinant of favorable outcome in severe mushroom-related hepatotoxicity	[46]
Lu et al., 2022	China (Qingdao)	To report successful treatment of *A. fuliginea* poisoning using hemoperfusion or continuous hemofiltration	Case series (n = 4)	Low	4 hospitalized patients with *A. fuliginea* poisoning (liver and kidney damage)	Symptomatic supportive care combined with hemoperfusion or continuous hemofiltration resulted in successful recovery in all four reported patients	Demonstrates the clinical utility of extracorporeal filtration techniques in managing multi-organ involvement in *A. fuliginea* poisoning	[47]
Huddam et al., 2021	Turkey (Mugla)	To report the use of medium cut-off membrane hemodialysis in *A. phalloides* intoxication	Case report (n = 2)	Low	2 patients with *A. phalloides* intoxication and hepatorenal involvement	Hemodialysis with MCO membrane improved liver and kidney function in both patients; patient 1 recovered fully; patient 2 died despite renal recovery due to *Acinetobacter* sepsis	Introduces MCO membrane hemodialysis as a novel extracorporeal tool for amatoxin poisoning, with renal and hepatic benefits but susceptibility to infectious complications	[48]
Li et al., 2021	China (Nanchang)	To report VA-ECMO combined with multimodal extracorporeal support in mushroom poisoning-induced cardiac failure	Case report (n = 1)	Low	56-year-old male with severe myocardial damage, MOD, circulatory failure, and recurrent malignant arrhythmia	VA-ECMO combined with hemoperfusion, plasma exchange, and CRRT; cardiac rhythm stabilized 3 h post-ECMO; heart function recovered on day 6	Demonstrates the feasibility of VA-ECMO as a rescue therapy for mushroom poisoning-induced refractory cardiogenic shock and cardiac arrest	[49]
Vetter, 2023	Not stated	To review the indications and evolution of liver transplantation in amatoxin poisoning since the mid-1990s	Narrative review	Low	Not specified	Liver transplantation justified when PT is very prolonged with metabolic acidosis, hypoglycemia, and elevated serum ammonia; may be orthotopic or partial; established as a practical option since the mid-1990s	Defines the clinical criteria and historical context for liver transplantation as the definitive intervention in refractory amatoxin-induced liver failure	[38]
Canbaz et al., 2025	Turkey	To evaluate outcomes of emergency liver transplantation for acute liver failure following mushroom intoxication	Retrospective cohort study (2008–2023)	Moderate	26 adult patients who underwent emergency liver transplantation for mushroom poisoning-induced acute liver failure	Overall survival rate: 69.2%; higher MELD scores and need for retransplantation associated with increased mortality; initial laboratory parameters at admission did not significantly differ between survivors and non-survivors	Supports timely referral to transplant centers and highlights MELD score and retransplantation need as key mortality predictors	[50]
Janatolmakan et al., 2022	International (multi-study)	To estimate pooled mortality and liver transplantation rates in mushroom poisoning through systematic review and meta-analysis	Systematic review and meta-analysis (33 studies)	Moderate–High	Patients with mushroom poisoning across 33 included studies; 16 transplanted patients	Mortality rates ranged 0–40%; pooled mortality ~2.87%; 16 transplanted patients, 14 survived; early referral to specialized centers and timely therapy may reduce transplantation need	Provides the highest-level evidence for liver transplantation survival benefit and endorses early specialized care to reduce transplantation necessity	[51]
Zhang et al., 2022	China (multi-center)	To evaluate the adjunctive effect of *Ganoderma lucidum* on clinical outcomes in acute mushroom poisoning with liver injury	Retrospective cohort study (May 2016–May 2021; 7 centers)	Moderate	61 adult patients with acute mushroom poisoning and liver injury	*G. lucidum* group: shorter hospital stay (6.69 ± 3.98 vs. 9.27 ± 5.30 days; *p* = 0.034), lower costs (16,336 vs. 27,540 CNY; *p* = 0.020), fewer patients requiring blood purification >48 h (30% vs. 69.23%; *p* = 0.027)	Supports G. lucidum as a promising low-cost adjunctive agent that may shorten hospital stay and reduce the burden of extracorporeal detoxification	[55]
Zuker-Herman et al., 2021	Australia (Heidelberg)	To report successful treatment of *A. phalloides* intoxication with intravenous rifampicin	Case report (n = 2)	Low	2 patients with acute *A. phalloides* poisoning following mushroom ingestion	Both patients successfully treated with intravenous rifampicin; no details on adverse effects reported	Provides preliminary clinical evidence for rifampicin as a potentially effective pharmacological option in amatoxin poisoning	[56]
Xing & Zhu, 2021	China (Shanghai)	To report the off-label use of etanercept (TNF-α blocker) in mushroom α-amanitin-induced liver injury	Case report (n = 2)	Low	2 patients with α-amanitin-induced liver injury and gastrointestinal symptoms at admission	Both patients treated with etanercept under close laboratory monitoring; TNF-α implicated in liver injury aggravation and inflammatory cascade; therapeutic efficacy suggested but safety evidence remains limited	Introduces TNF-α blockade as a novel experimental approach for amatoxin hepatotoxicity, with potential but unproven safety in liver-injured patients	[57]
Min et al., 2022	South Korea (Yangsan)	To report a case of Russula subnigricans poisoning initially misdiagnosed as NSTEMI due to rhabdomyolysis-induced cardiac involvement	Case report (n = 3; one index patient + two family members)	Low	64-year-old man with chest pain, elevated troponin I, nausea, vomiting, and myalgia; two family members with rhabdomyolysis	Initial misdiagnosis as NSTEMI; conservative treatment with fluid resuscitation was successful; all three patients discharged without complications	Highlights the diagnostic challenge of mushroom-induced rhabdomyolysis mimicking acute coronary syndrome and supports fluid resuscitation as effective first-line management	[58]
Zhong et al., 2023	China (Chuxiong)	To report a case of *A. neoovoidea* intoxication presenting with acute renal injury successfully managed with supportive care and blood purification	Case report (n = 1)	Low	Single patient with *A. neoovoidea* intoxication presenting with nausea, vomiting, oliguria, and acute renal function injury	Successful recovery after symptomatic supportive treatment combined with bloods purification therapy; patient discharged without further complications	Illustrates the nephrotoxic potential of *A. neoovoidea* and the efficacy of blood purification in managing acute renal involvement in non-classic *Amanita* poisoning	[59]

## Data Availability

The original contributions presented in this study are included in the article. Further inquiries can be directed to the corresponding author.
